# Is there any cardioprotective role of Taurine during cold ischemic period following global myocardial ischemia?

**DOI:** 10.1186/1749-8090-6-31

**Published:** 2011-03-18

**Authors:** Mehmet A Sahin, Orhan Yucel, Adem Guler, Suat Doganci, Artan Jahollari, Faruk Cingoz, Sıddık Arslan, Mehmet Gamsizkan, Halil Yaman, Ufuk Demirkilic

**Affiliations:** 1 Gülhane Military Medical Academy, Department of Cardiovascular Surgery, 06010, Etlik, Ankara, Turkey; 2 Gülhane Military Medical Academy, Department of Thoracic Surgery, 06010, Etlik, Ankara, Turkey; 3 Gazi University, Faculty of Commerce and Tourism Education, Department of Computer Applications Training, 06830, Gölbaşı, Ankara, Turkey; 4 Gülhane Military Medical Academy, Department of Pathology, 06010, Etlik, Ankara, Turkey; 5 Gülhane Military Medical Academy, Department of Biochemistry, 06010, Etlik, Ankara, Turkey

## Abstract

**Background:**

The aim of the present study was to investigate the cardioprotective effect of Taurine on the donor hearts during cold ischemic period.

**Methods:**

32 rats were divided into four groups (sham, taurine, ischemia, treatment group, 8 rats in each). All rats were fed with rat food for three weeks. Taurine and treatment groups were given a 200 mg/kg/day dose of Taurine by oral gavage besides rat feed. Cardiectomy was performed in all rats after three weeks. In ischemia and treatment groups, harvested hearts were kept in 0.9% sodium chloride at +4 degrees C for 5 hours. Tissue samples were taken from left ventricle in all groups. These samples were evaluated by histopathologic and biochemical examination.

**Results:**

In the present study results of the biochemical and histopathological examination reveals the protective effects of Taurine. As a marker of lipid peroxidation, Malondialdehyde (MDA) levels in ischemia group were significantly higher than both Sham and Taurine groups. MDA values were recorded; 3.62 ± 0.197 in the sham group, 2.07 ± 0.751 in the Taurine group, 9.71 ± 1.439 in the ischemia group and 7.68 ± 1.365 in the treatment group. MDA levels decreased in treatment group. (p < 0.05) In accordance with MDA findings, while superoxide dismutase and glutathione peroxidase levels decreased in ischemia group, they increased in treatment group. (p < 0.05) There was no differences in Catalase (CAT) enzyme level between treatment and ischemia group (p = 1.000). CAT level results were recorded; 7.08 ± 0.609 in the sham group, 6.15 ± 0.119 in the Taurine group, 5.02 ± 0.62 in the ischemia group, and 5.36 ± 0.384 in the treatment group. Less intracellular edema and inflammatory cell reaction were observed in histologic examination in favor of treatment group. (p < 0.01)

**Conclusion:**

Taurine decreased myocardial damage during cold ischemic period following global myocardial ischemia.

## Background

Maintaining cardiac functions in explanted hearts within ischemic time needs good preservation. Hypoxic, hypothermic, cardioplegic arrest followed by cold transport is a common procedure for preservation of explanted hearts. This procedure is the main practical method used for preserving donor organs in many transplant centers [1].

Unfortunately, there is no perfect protection method for donor organs currently. With the increase in the ischemic time following explantation, tissue and the organ damage are almost inevitable. Organ functions can be improved by minimizing the myocardial function during ischemia. For this purpose many studies have been performed to prolong this ischemic time or protect the organs in this deleterious process.

Taurine (2-amino ethane sulfonic acid) is a potent antioxidant agent. It is shown that Taurine has beneficial effects on myocardial ischemia-reperfusion injury,[2-6] cardiomyopathy, congestive heart failure[7,8] and pulmonary edema [9].

The aim of this study was to investigate the cardioprotective role of oral Taurine administration in explanted ischemic hearts which were kept in cold isotonic solution for 5 hours.

## Methods

This study was conducted in compliance with "Principles of Laboratory Animal Care" determined by National Institutes of Health (National Institutes of Health, publication No: 85-23, revised 1985). The experiment and animal care protocol was approved by Gülhane Military Medical Academy local ethical committee of animals use.

### Animals

Thirty-two male rats (Rattus norvegicus) approximately 17-19 weeks of age and weighing 330 ± 10.25 g were used in this study. Animals were obtained from licensed suppliers and quarantined for a minimum of seven days before entering into the study. All animals were maintained in the Gülhane Military Medical Academy fully accredited Animal Care Facility under the rules and regulations of the Care and use of Laboratory animals.

### Study Design

**Figure 1:**
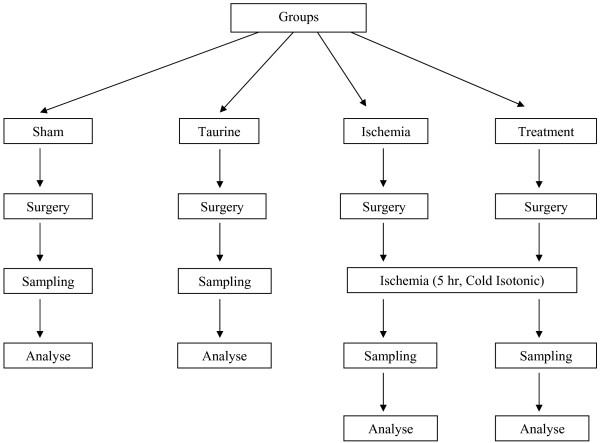
**Consort diagram of the study**.

Following quarantine period, rats were put in wire cages for three days before the study. They were fed with standard rat feed (Bil-Yem Food Industry, Yenikent-ANKARA/TURKEY) and tap water was placed near the cage. Four groups, including randomly chosen 8 rats in each of them, were constituted. Sham group rats were fed with standard rat feed. Taurine group rats had additional Taurine to the feed. Ischemia group rats were fed with standard feed and ischemia was established. Treatment group rats were fed with Taurine and ischemia was established. Taurine was given with dose 200 mg/kg/day via oral gavage method in addition to standard feed to provide standardization. The primary characteristics of the groups were shown in Table 1. All animals were cared for three weeks before the experimental procedures. The consort diagram of the study was shown in Figure 1.

**Table 1:** Primary characteristics of groups

Groups	(n)	Nutrition	Nutrition Time	Sampling Time
Sham	8	Standard feed	Three weeks	Immediately after cardiectomy
Taurine	8	Standard feed+Taurine	Three weeks	Immediately after cardiectomy
Ischemia	8	Standard feed	Three weeks	5 hours after cardiectomy
Treatment	8	Standard feed+Taurine	Three weeks	5 hours after cardiectomy

### Anesthesia and Surgery

Animals were anesthetized with intraperitoneal ketamine (75 mg/kg) and xylazine (10 mg/kg). Heparin (5 IU/g body weight) was given intraperitoneally for 30 minutes before explantation of heart to prevent the microembolic events. Chests were scrubbed with alcohol and betadine. Median sternotomy was performed. Aorta was cannulated and inferior vena cava was cut. Cross clamp was placed to the aorta and plegisol (Plegisol Cardioplegic Solution, Sanofi Synthelabo Industry, Turkey) infused to the heart to wash the intracardiac vascular bed, while blood was removing from inferior vena cava. Hearts were removed after cardiac arrest. In sham and Taurine groups, following the explantation of the heart, samples were immediately taken for analysis from left ventricle. However, in Ischemia and Treatment groups explanted hearts were kept in a cold solution (0. 9% isotonic solution, +4 degrees C). For these groups, samples from left venticles were taken after 5 hours of cold ischemic period.

### Tissue Preparation

Biochemical samples were placed in liquid nitrogen in polypropylene tubes and kept in deep freeze (-80 degrees C). Histopathological samples were fixed in 10% formaldehyde.

### Histopatological Analysis

The paraffin-embedded tissues were sectioned and stained with hematoxylin-eosin. The histological slides were evaluated by a pathologist who was blinded to experiment protocol. The following morphological criteria were used to determine the histopathological damage: score 0, no damage; score 1 (mild), interstitial edema and focal necrosis; score 2 (moderate), diffuse myocardial cell swelling and necrosis; score 3 (severe), necrosis with the presence of contraction bands, neutrophil infiltration and the capillaries were compressed; and score 4 (highly severe), widespread necrosis with the presence of contraction bands, neutrophil infiltration, compressing capillaries and hemorrhage [10,11].

### Biochemical analysis

The frozen tissues were homogenized at a concentration of 100 mg tissue per ml of 25 mM phosphate buffer (pH 7.4) on an ice cube using a homogenizer (Heidolph Diax 900; Heidolph Electro GmbH, Kelheim, Germany) at a setting of 8 (out of 10) for 30-s bursts. The homogenates were centrifuged for 10 min at 2500 *g*, and the pellet (cellular debris) discarded. The supernatant was allocated into 2-3 separate tubes and used for biochemical assays.

### Tissue lipid peroxidation

The lipid peroxidation level was measured by using Draper and Hadley's Method [12]. This method uses spectrophotometric measurements of the color produced during the reaction of thiobarbituric acid with malondialdehyde (MDA). The absorbance of the final solution was measured at 532 nm, and MDA levels were expressed as MDA (mmol)/protein (g).

#### Superoxide dismutase (SOD)

SOD level was assayed using the nitroblue tetrazolium (NBT) method of Sun et al. [13]. NBT was reduced to blue formazan by superoxide which has a strong absorbance of 560 nm. One unit (U) of SOD is defined as the amount of protein that inhibits the rate of NBT reduction by 50%. The calculated SOD level was expressed as SOD (U)/protein (g).

#### Glutathione peroxidase (GPx)

GPx level was measured by using the method described by Paglia and Valentine in which GPx level was coupled with the oxidation of NADPH by glutathione reductase [14]. The oxidation of NADPH was spectrophotometrically followed up at 340 nm at 37 degrees C. The absorbance at 340 nm was recorded for 5 min. The level was the slope of the lines (mmol) of oxidized NADPH/min. GPx level was presented as GPx (U)/protein (g).

#### Catalase (CAT)

CAT level was determined spectrophotometrically, by direct measurement of the decrease of light absorption at 240 nm caused by the decomposition of hydrogen peroxide by Catalase [15].

### Statistical Analysis

SPSS for Windows Version 15.00 (Statistical Package for the Social Sciences, SPSS Inc., Chicago, IL., USA) package program was used for all statistical analyses and measurements. Compliance of biochemical measurement values to normal distribution was examined graphically and statistically through the Shapiro-Wilk test. Among the variables, it was determined that MDA and SOD variables were not in compliance with normal distribution. For definitive statistics, mean values were given with the average standard deviation. One way variance analysis (One Way ANOVA) was used for comparison of GPx and CAT measurements; and Kruskal-Wallis variance analysis was applied for MDA and SOD parameters. The Bonferroni and Mann-Whitney U test was used for bilateral comparisons within the groups. p < 0.05 value was accepted as statistically significant.

## Results

### Biochemical examination results

#### MDA Results (nmol/g)

**Figure 2:**
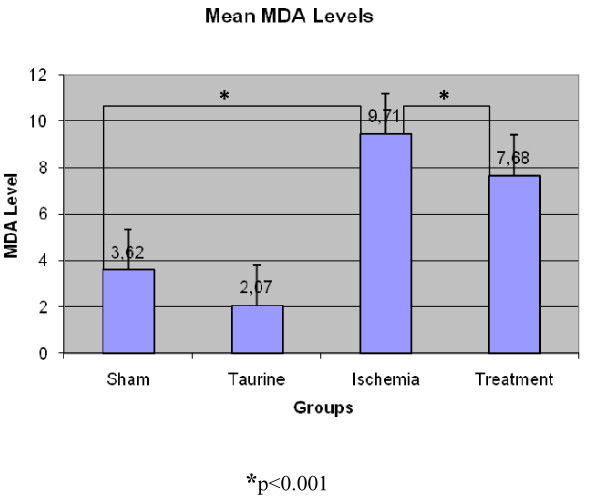
**MDA levels in rat myocard tissue**.

MDA values were recorded accordingly; 3.62 ± 0.197 in the sham group, 2.07 ± 0.751 in the Taurine group, 9.71 ± 1.439 in the ischemia group and 7.68 ± 1.365 in the treatment group. (Figure 2) The bilateral difference between all groups was found to be statistically significant (p < 0.05). When average values were examined, the lowest value of MDA level was recorded in Taurine group and the highest value was recorded in the ischemia group.

#### SOD Results (U/g)

**Figure 3:**
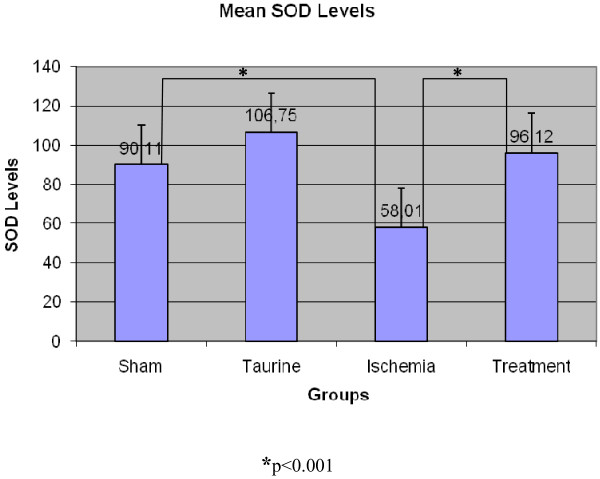
**SOD enzyme levels in rat myocard tissue**.

SOD level was recorded accordingly; 90.11 ± 5.222 in the sham group, 106.75 ± 3.449 in the Taurine group, 58.01 ± 4.244 in the ischemia group, and 96.12 ± 7.886 in the treatment group (Figure 3). The difference between the sham group and treatment group was statistically insignificant and bilateral differences between other groups were found statistically significant. SOD values that decreased in the sham Group were increased in the Treatment group to which Taurine was administered, and this difference between the ischemia group and the treatment group was found to be statistically significant (p < 0.001). The lowest SOD value was observed in the ischemia group and the highest SOD value was recorded in the Taurine group.

#### GPx Results (U/g)

**Figure 4:**
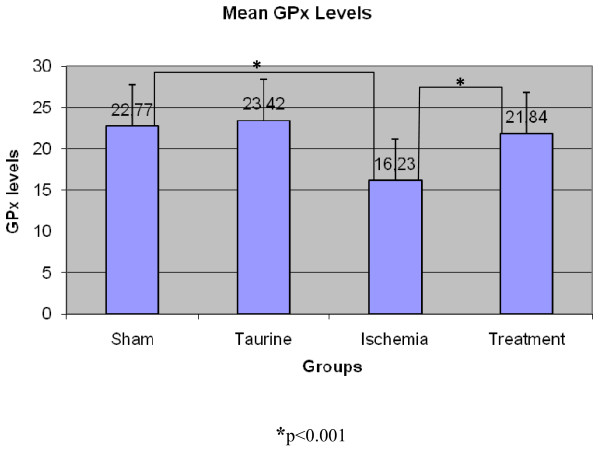
**GPx enzyme levels in rat myocard tissue**.

GPx values were recorded accordingly; 22.77 ± 1.308 in the sham group, 23.42 ± 2.031 in the Taurine group, 16.23 ± 1.131 in the ischemia group, and 21.84 ± 3.298 in the treatment group (Figure 4). The difference between the ischemia and the treatment groups and the ischemia and the sham groups was found to be statistically significant (p < 0,001).

#### CAT Results (KU/g)

**Figure 5:**
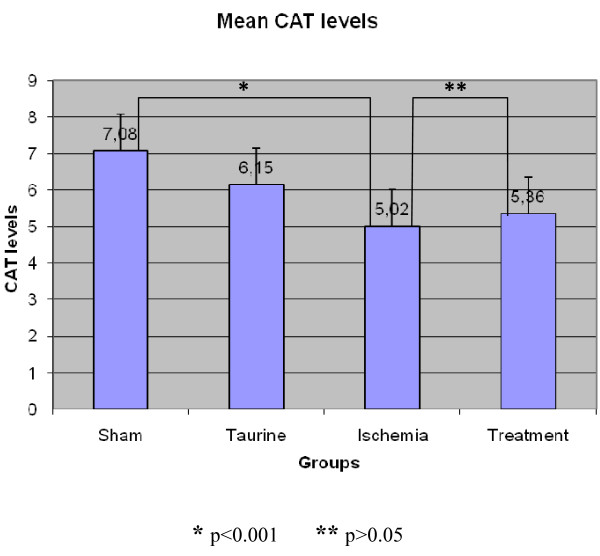
**CAT enzyme levels in rat myocard tissue**.

CAT level results were recorded accordingly; 7.08 ± 0.609 in the sham group, 6.15 ± 0.119 in the Taurine group, 5.02 ± 0.62 in the ischemia group, and 5.36 ± 0.384 in the treatment group. (Figure 5) The difference between ischemia and treatment groups was found to be statistically insignificant (p > 0.05), and bilateral differences between the other groups were found significant. When compared to the sham group, there was not a significant increase in ischemia group (p = 1,000).

### Histopathological results

**Figure 6:**
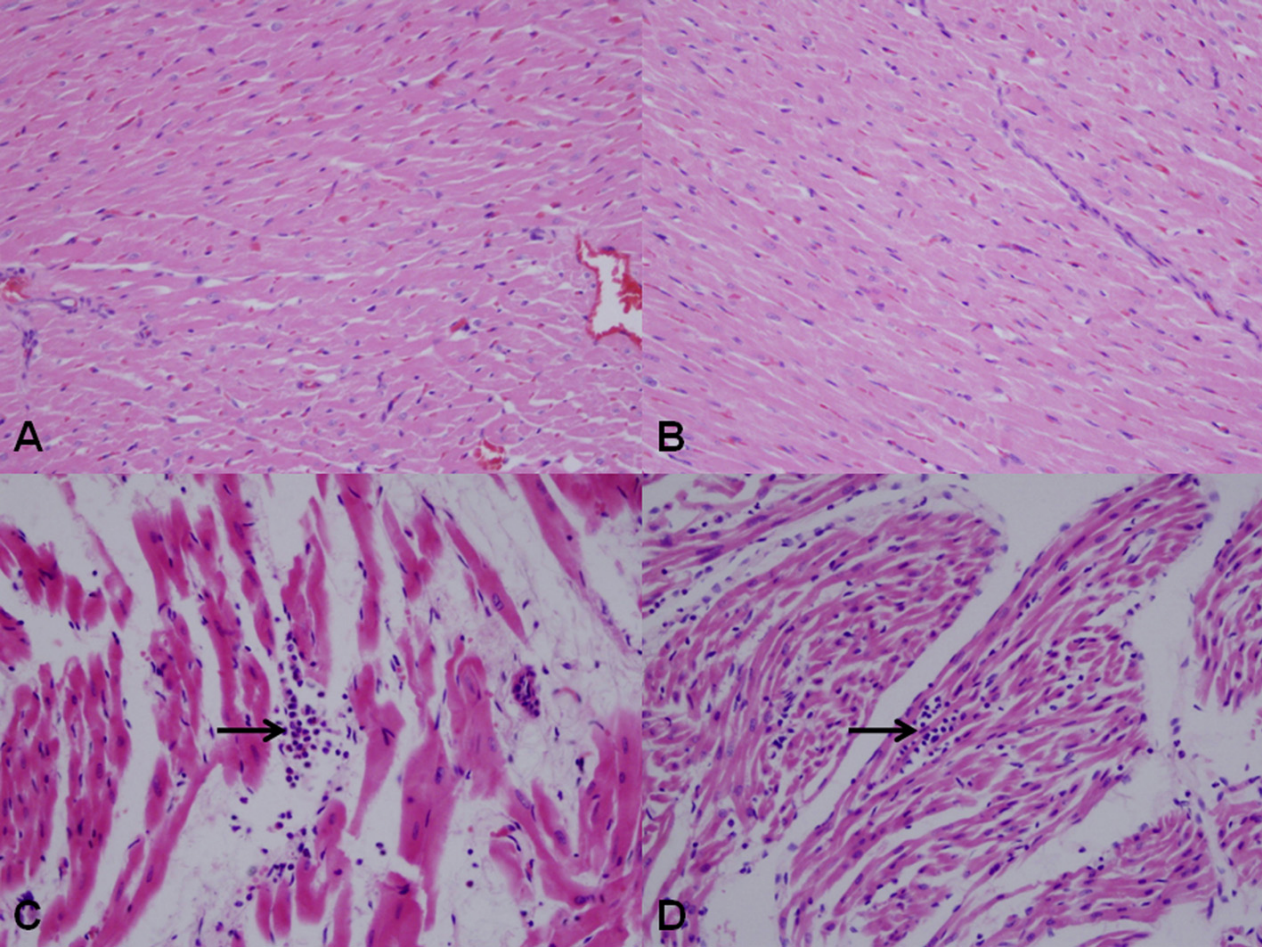
**Histopathological view of the myocardial tissue samples from each group**. Muscle fibers in normal appearance are seen in sham (A) and Taurine (B) groups (HEx400). Muscle fibers are separated in ischemia group due to interstitial edema and muscle fibers are in more acidophilic appearance. PMN leukocyte infiltration between the muscle fibers is seen (arrow) (C) (HEx400). Distribution of muscle fibers in treatment group seems better preserved when compared to ischemia group. Inflammatory cell infiltration is observed in the arrowed area. (D) (HEx400).

Muscle fibers in sham and Taurine groups were in normal limits. (Figure 6A and 6B) In ischemia group, myofibrils were relatively insignificant with intense acidophil cytoplasm, pyknotic-dark or light nucleus. Besides, the muscle fibers were disorganized and swelling. They were separated due to interstitial edema. PMN leukocyte groups were observed in the vessel walls or by penetrating into the connective tissue. (Figure 6C) Degranulation was also observed from mast cells to the connective tissue. In the treatment group, the distribution of the muscle fibers was better preserved when compared to ischemic group. In addition, the level of interstitial edema and inflammatory cell infiltration was lower than the ischemia group. (Figure 6D) The mean histopathological damage in treatment group and ischemia group were scored 1.8 ± 0.8 vs 2.3 ± 0.7. (p < 0.01)

## Discussion

The primary mission during ischemic period is to provide micro-vascular, cellular and functional integrity of the myocardium as much as possible. This needs cellular energy. Heart should be immediately stopped after placing cross clamp in order to protect cardiac energy storages. Cold preservation solutions are commonly used protective media to keep the donor organs in good condition during whole ischemic time. Good preservation prevents ischemic damages and reperfusion injury and minimizes cellular damage [16].

Taurine is a semi-essential amino acid that supports neurological and musculoskeletal system development. Taurine comprises 50% of the cardiac free amino acid pool and is present in the myocardial tissue in the concentration of 11-38 μM/g [5]. It plays an important role in the regulation of sodium, potassium, calcium, and ion flow along with cardiac contractility, regulation of membrane excitability, osmolality and the volume content [17,18]. Diet is the main source of Taurine in humans. Taurine occurs naturally in food, especially in seafood and meat. The mean daily taurine intake for adult human has been estimated between 40-400 mg [19]. Although various doses of Taurine (25 mg/kg/day to 6 g/day, p.o. or i.v.) in human and animal studies reported,[19,20] we preferred to use a dose of 200 mg/kg/day administered orally (with the help of gavage).

There is a strong connection between Taurine excretion levels and ischemic heart disease mortality [21]. It is shown that preoperative Taurine infusion decreases reperfusion injury in coronary artery bypass surgery [22]. Taurine that was given as a dietary supplement to the rats' meal before inducting myocardial infarction decreases infarct size and improves heart functions after myocardial infarct [23].

Some structural changes occur in the myocardial cells during the cold ischemic period. High energy phosphate synthesis decreases as a result of decreasing oxidative phosphorilation. Na^+^-K^+^-ATP-ase pump in the cell membrane deteriorates and the energy storage of the cell decreases. Na^+ ^and Ca^2+ ^ions accumulate in the cell. The accumulation of Ca^2+ ^ions in the cell results in cytotoxicity and subsequently antioxidant enzyme levels are reduced in cells. Ultimately; swollen cells, extracellular edema, acidosis, calcium accumulation, and endothelial damage occur. This situation makes myocardial cell more sensitive to oxidative damage during reperfusion period [24-26]. This study histopathologically and biochemically proves that taurine administration decreases the myocardial damage occured during the cold ischemic period. In this study, significant swollen cell and intense inflammatory reaction were observed in the donor hearts preserved in +4 degrees C and exposed to ischemia. Swollen cell number and inflammatory reaction were much less in the treatment group than others. It was found that Taurine decreases histopathologic changes that might occur during cold ischemic time. (Figure 6)

Free oxygen radicals are produced in all body cells in a limited number under normal conditions and are neutralized by endogenous anti oxidants such as superoxide dismutase, glutathione peroxidase and catalase (Scavenging Enzyme Systems). Free oxygen radicals cause tissue damage through the peroxidation of the lipids present in the cell membranes.

Increasing lipid peroxidation might be used as a sign of the tissue damage caused by free oxygen radicals. MDA is the final product of lipid peroxidation. Measurement of the MDA level in serum might be used as an indicator of tissue damage caused by in vivo free oxygen radicals [27,28]. Kaplan and colleagues showed that taurine deficiency caused an increase in MDA levels. In our study we also found that MDA values were very high in the ischemia group, and decreased in the treatment group (p < 0.05).

Cells are highly affected by oxidative damage if antioxidant enzymes decrease in the tissue. Superoxide dismutase enzyme system is the first and the most important defense mechanism of the body against free oxygen radicals [29]. If there is enough superoxide dismutase activity, cell damage occurs at minimum level. In a study by Bolcal et al,[30] cardioprotective role of antioxidant medications was researched. In this study there were protective increases in SOD and GPx levels and a decrease in MDA levels were reported. In our study, although we studied Taurine as antioxidant medication, there were similar results. SOD enzyme levels in the ischemia group decreased when compared to the sham group, but increased in the Taurine administered treatment group. This increase is found to be statistically significant (p < 0, 05) and this raising in the treatment group is found to be close in the sham and Taurine group.

Catalase is an antioxidant enzyme. It degrades hydrogen peroxide (H_2_O_2_) to oxygen and water. Catalase acts together with GPx in that process. H_2_O_2 _concentration is diminished by Catalase [31,32]. In our study, when Catalase levels were examined, no statistically significant difference was found between ischemia and treatment groups. The probable mechanism of this could be uninvolvement of the cells with high CAT enzyme levels in the process. The CAT enzyme levels were realized to have been decreased probably due to the processed hydrogen peroxides. There was not a remarkable difference between ischemia and treatment group since the treatment group did not have high CAT level obtained by Taurine.

## Study Limitations

Main limitation of this study is the administration way of Taurine and its clinical impact. In the literature there are many studies with very large range of administration periods (5 min before ischemia to 7 weeks before the study). Also there are very different study doses of Taurine. In our study we tried to use a mean value and duration according to the literature. Although the Taurine cardiac effects are well known there are limited reports related to the ischemia of the donour hearts. It is not practical to use Taurine three weeks before an unpredicted ischemia, but our aim was only to show if there is any beneficial effect of supplemental Taurine in such situations. We think that it can play an important role in heart explantation operations. Detailed protocols of Taurine usage prior to explantation ischemia has yet to be established and different administration ways and dosages just before the predicted ischemia may be subject of other studies.

## Conclusion

This study demonstrated that Taurine decreased ischemic cellular damage in rat hearts that were kept under ischemic and cold circumstances for 5 hours. We believe that these beneficial effects of Taurine may be related to its antioxidant effect.

## List of abbreviations

CAT: Catalase; GPx: Glutathione peroxidase; H_2_O_2_: Hydrogen peroxide; MDA: Malondialdehyde; NBT: Nitroblue tetrazolium; SOD: Superoxide dismutase; SPSS: Statistical Package for the Social Sciences; U: Unit

## Competing interests

The authors declare that they have no competing interests.

## Authors' contributions

MAS, OY, AG and UD were both involved in the conception of the study design as well as drafting and revising the article. SD, AJ and FC contributed to the surgical procedures. MG and HY were involved in acquisition of pathologic and biochemical data. SA was involved in statistical analysis of data. All authors have approved the manuscript.
